# Application-driven pedagogical knowledge optimization of open-source LLMs via reinforcement learning and supervised fine-tuning

**DOI:** 10.3389/frai.2026.1851993

**Published:** 2026-07-01

**Authors:** Navan Preet Singh, Xiaokun Wang, Anurag Garikipati, Madalina Ciobanu, Qingqing Mao, Ritankar Das

**Affiliations:** 1 Department of Research and Development, Forta, Houston, TX, United States; 2 East China Normal University, Shanghai, China; 3 Department of Research and Development, Incept Labs, Houston, TX, United States; 4 Department of Research and Development, Titan Holdings, San Francisco, CA, United States

**Keywords:** agentic AI, digital accessibility, LLM application, open-source, pedagogical content knowledge, reinforcement learning, supervised fine-tuning

## Abstract

We present a multi-stage optimization strategy combining reinforcement learning (RL) and supervised fine-tuning (SFT) to enhance the pedagogical knowledge of Large Language Models (LLMs), thereby providing a technically grounded example of how open-source pedagogical LLMs can be optimized for deployment in diverse educational settings, including institutions with constrained resources. Our approach produces EduQwen 32B-RL1, EduQwen 32B-SFT, and EduQwen 32B-SFT-RL2: (1) first-stage RL optimization implementing progressive difficulty training, focusing on challenging examples, and employing extended reasoning rollouts to facilitate adaptive scaffolding, prioritizing pedagogical steering over direct answer provision; (2) SFT that leverages the RL-trained model to synthesize high-quality training data with difficulty-weighted sampling; and (3) optional second-stage RL refinement. This application-driven family of open-source pedagogical LLMs, built on a dense Qwen3-32B backbone, achieves 96.52% accuracy on the Cross-Domain Pedagogical Knowledge (CDPK) Benchmark, establishing new state-of-the-art (SOTA) performance on the CDPK subset of the interactive Pedagogy Benchmark Leaderboard as of March 2026, with a 5.97 percentage points accuracy gain over the then-reported Gemini-3 Pro's score (previous leader with 90.55% accuracy), under the respective documented evaluation protocols. Critically, our 32-billion-parameter models demonstrate that domain-specialized optimization of mid-sized open-source LLMs can outperform much larger general-purpose systems on pedagogical knowledge benchmarks, indicating a scalable technical approach for wider AI deployment and potential pedagogical support, while preserving the transparency, customizability, and cost-efficiency required for responsible educational AI development, with deployment effectiveness depending on teacher judgment, learner context, and authentic instructional integration across diverse global learning environments.

## 1 Introduction

Large Language Models (LLMs), driven by deep learning algorithms, are sophisticated systems designed to synthesize knowledge and engage in nuanced natural conversations. These models demonstrate remarkable proficiency in generating coherent, contextually rich output, a capability that has positioned them as transformative instruments across various high-stakes industries (e.g., finance, law, and medicine). While premium proprietary LLMs (e.g., GPT-5, Claude, and Gemini) have historically set general state-of-the-art (SOTA) performance benchmarks (Minaee et al., 2024; Hadi et al., 2023), advanced open-source LLMs—such as Mistral 3, DeepSeek-R1, and Qwen3—offer significant advantages in research and development due to their publicly accessible architectures and algorithmic transparency (Maharjan et al., 2024; Manchanda et al., 2025). A key downside of commercial models is the difficulty and cost associated with fine-tuning, owing to their opacity as well as significantly larger size compared to smaller, specialized open-source models (Liao and Vaughan, 2023; Wu et al., 2024).

In the field of pedagogy, LLMs offer the potential for scalable support for personalized instruction; while it is currently cost-prohibitive, one-on-one tutoring delivers significantly better educational outcomes (Bloom's Two-Sigma Problem) (Bloom, 1984). Open-source LLMs are particularly attractive for implementation in education; when compared with commercial models, they allow for easier fine-tuning for specific requirements (e.g., pedagogy), which enables the creation of more affordable customized tools that can adapt dynamically to individual learner needs (Perczel et al., 2025; Yan et al., 2024). The opacity of commercial, proprietary LLMs limits external scrutiny and customization (Liao and Vaughan, 2023), presenting a significant barrier to effectively addressing pedagogical steering and bias mitigation. Additionally, commercial models often incur high, usage-based fees and restrict control over data and deployment; the commercial models can also update without notice, leading to development instability. Conversely, the full control and transparency afforded by open-source models facilitate the community-wide scrutiny and domain-specific engineering necessary for transparency, ethical adherence, and robust accountability (NTIA, 2024). These considerations are especially critical for educational applications, where deployment at scale directly impacts learning outcomes for diverse student populations, particularly in under-resourced settings where proprietary model costs create access barriers. Actualizing the potential of AI tools in education, however, requires acknowledging that real-world classroom impact depends on teacher judgment, learner context, and sustained pedagogical engagement (Rind, 2026).

LLMs can open the door for global personalized education, however, currently these models are typically optimized to prioritize immediate helpfulness (Puech et al., 2024). This leads to a misalignment with the concept of guided learning in which the goal is not to provide the student with the answer, but rather help them get to the answer themselves. This structural gap underscores the importance of evaluating LLMs with specialized frameworks that explicitly distinguish between content knowledge (factual understanding) and pedagogical knowledge (strategic teaching capacity). A recent study introduced The Pedagogy Benchmark—a publicly available dataset of over 1,100 teacher exam questions—demonstrating that commercial and open-source LLMs achieve wide-ranging pedagogical knowledge scores from 21 to 91%; interactive leaderboards are published in order to transparently compare the performance, cost, and reasoning abilities on pedagogical tasks of LLMs from a growing list of 150 (AI for Education, 2026; Lelièvre et al., 2025). Despite the growing availability of pedagogical benchmarks and the demonstrated potential of open-source LLMs, no prior work has systematically investigated whether targeted multi-stage optimization combining RL and SFT can close the performance gap between open-source and proprietary models on Cross-Domain Pedagogical Knowledge (CDPK) tasks, nor demonstrated that mid-sized open-source models can be brought to match or exceed frontier proprietary systems on pedagogical knowledge benchmarks. This gap is consequential: without technically strong, openly accessible pedagogical models, equitable deployment of AI-based educational support in resource-constrained institutions remains out of reach. Closing this performance gap requires more than just technical scaling; it requires aligning the models with established pedagogical principles.

Theoretical Grounding of Pedagogical Knowledge. To rigorously evaluate and optimize LLMs for educational settings, it is necessary to ground model behavior in established learning sciences. In this study, we distinguish between standard content knowledge, which is the factual mastery of a subject, and pedagogical content knowledge (PCK), which encompasses the strategic understanding of how to make that content comprehensible to learners (Shulman, 1986). A robust pedagogical model must exhibit instructional decision-making that adapts representations to the learner's specific needs (Shulman, 1987). This distinction is operationalized in the CDPK Benchmark, which explicitly tests strategic pedagogical reasoning rather than content knowledge alone (Lelièvre et al., 2025). At the core of this adaptive interaction is the concept of scaffolding, derived from Vygotsky's Zone of Proximal Development, which emphasizes supporting learners toward independent understanding rather than directly providing answers (Vygotsky, 1978). In automated tutoring, this is achieved through human-inspired conversational scaffolding and student-centered dialogue (Nye et al., 2014), alongside high-quality formative feedback. Effective formative feedback functions not as summative answer-confirmation, but as ongoing, task- and process-level interaction designed to reduce the gap between a learner's current understanding and their learning goals (Black and Wiliam, 1998; Hattie and Timperley, 2007). Because most foundational LLMs are optimized for immediate helpfulness, they inherently conflict with these principles of guided learning. Consequently, realizing the potential of AI tutor-like systems requires intentional pedagogical steering, i.e., aligning the model's reward systems with instructional decision-making and adaptive tutoring principles rather than mere factual accuracy (Puech et al., 2024). Acknowledging these theoretical foundations also requires recognizing that benchmark performance on pedagogical knowledge questions captures only one dimension of pedagogical capacity; actual classroom impact depends on teacher judgment, learner epistemic orientation, and sustained deployment in authentic instructional contexts (Rind, 2026; Rind et al., 2026).

To directly address the the performance gap between open-source and proprietary models on pedagogical knowledge tasks, we strategically enhanced the cross-domain pedagogical knowledge of open source models, as tested on the CDPK Benchmark subset of The Pedagogy Benchmark. Our work leverages the transparency and fine-tuning capacity of open-source models through a multi-stage optimization combining reinforcement learning (RL) and supervised fine-tuning (SFT), thereby advancing the development of trustworthy and accessible AI tools with potential for tutoring applications. In this study, we present a model optimization strategy that establishes new state-of-the-art (SOTA) performance on the CDPK Benchmark. Remarkably, our approach, which applies advanced model training and robust inference-time compute techniques to the dense 32-billion parameter open-source model Qwen3-32B, achieves up to 96.52% accuracy, representing the highest reported accuracy on the CDPK subset on the interactive Pedagogy Benchmark leaderboard as of March 2026 (AI for Education, 2026). This includes significantly larger models, indicating that domain-specialized smaller models can achieve SOTA benchmark performance and, in this setting, match or exceed general-purpose large-scale systems on pedagogical knowledge benchmarks, while offering superior cost-efficiency and more practically deployable pathways for institutions with limited computational infrastructure.

This paper makes three contributions to educational applications of LLMs: (1) the development of EduQwen 32B-RL1, EduQwen 32B-SFT, and the optional third-stage model EduQwen 32B-SFT-RL2 as open-source pedagogical domain experts; (2) a multi-stage pipeline that combines pedagogically aligned RL with difficulty-weighted SFT using RL-generated synthetic data; and (3) empirical evidence that this approach achieves new SOTA performance on the CDPK Benchmark, surpassing the reported scores (March 2026) of much larger proprietary models while remaining practical for potential real-world educational deployment pending further validation. Our study is therefore positioned primarily as an application-driven contribution advancing the technical foundations for more widely accessible educational AI, demonstrating how targeted optimization can turn open-source LLMs into high-precision pedagogical knowledge systems that achieve SOTA benchmark performance on pedagogical knowledge tasks, while leaving real-classroom tutoring effectiveness as an open question for future empirical study. This concept could find future applicability in diverse global contexts including resource-limited educational settings.

## 2 Methodology

### 2.1 Benchmark evaluation

The evaluation of LLM performance was conducted exclusively using the CDPK Benchmark, to ensure domain-specific validation of pedagogical knowledge. The CDPK Benchmark is a specialized dataset designed to explicitly distinguish between content knowledge and strategic pedagogical knowledge (Lelièvre et al., 2025), which is crucial for informing the responsible and transparent deployment of LLMs in educational settings. The CDPK Benchmark comprises a publicly available collection of 920 teacher multiple-choice exam questions, ensuring a rigorous assessment of the models' capacity to support effective teaching practices in a standardized and openly comparable way.

Model performance was primarily evaluated by calculating accuracy—the percentage of correct responses against a ground truth answer key—a standard metric for static, ground-truth-based educational assessments. The final comparative performance of the tested open-source LLMs was assessed against the interactive Pedagogy Benchmark leaderboard (AI for Education, 2026). This methodology provides a transparent and standardized comparison of the developed platform against a growing list of open-source and commercial LLMs, crucial for benchmarking pedagogical LLM performance and informing decisions about which models are practically suitable for broader, cost-sensitive educational deployment.

### 2.2 Base model selection

We selected Qwen3-32B as our base model based on systematic evaluation of diverse open-source LLMs to determine the optimal foundation for pedagogical optimization, as illustrated by the performance on the CDPK Benchmark. Qwen3-32B, a dense model developed by Alibaba (Yang et al., 2025), was chosen for its strong baseline performance on the CDPK Benchmark and demonstrated amenability to advanced fine-tuning methods. The Qwen3-32B foundation model is a highly competitive open-source LLM derived from the Qwen3 series architecture, providing the transparency and flexibility that are particularly advantageous for educational deployment while maintaining computational efficiency through its dense 32-billion parameter architecture that can be realistically considered by institutions with constrained computational resources. We evaluated models spanning both dense and mixture of experts (MoE) architectures in preliminary experiments, finding that dense architectures proved more responsive to iterative optimization (Kim et al., 2025; Wang et al., 2024). This rigorous evaluation identified Qwen3-32B as an optimal base LLM for pedagogical specialization, forming the foundation upon which we construct EduQwen 32B-RL1 via RL optimization and EduQwen 32B-SFT via subsequent SFT as a technically feasible pathway toward deployable pedagogical models in varied educational contexts.

### 2.3 Model training

This paper proposes a multi-stage iterative training pipeline, RL-SFT-RL, to enhance the reasoning capabilities of LLMs in the pedagogy domain. The process begins with an initial or first round of RL, where a base model (Qwen3-32B) is trained to identify and focus on challenging problems. Subsequently, an SFT phase is introduced to refine the model's proficiency. Finally, an optional second round of RL is conducted to solidify the gains before considering downstream investigation in applied educational contexts. Complete training hyperparameters, hardware specifications, and prompt templates are detailed in the Supplementary material.

We note several details of our data handling and evaluation protocol relevant to interpreting the reported results. The pedagogy dataset was partitioned into training and test subsets, and the questions used to construct the RL and SFT training curricula were drawn from the training subset; final accuracies were computed on the test subset. Consistent with the study's focus on the effect of pedagogical optimization on model steering rather than on establishing leaderboard rankings *per se*, training and evaluation were conducted within the same benchmark distribution, and the optimization process was oriented toward the pedagogy benchmark. We did not perform an explicit semantic-overlap or deduplication analysis between the synthetic training examples and the benchmark items. Convergence was monitored throughout training via the reward signal rather than a separately held-out validation partition. To establish statistical validation, we provide 95% confidence intervals (CIs) for all evaluated model accuracies, and we additionally provide a two-tailed significance *p* value to evaluate the significance of the performance gains between the Qwen3-32B baseline and our final model tier.

#### 2.3.1 RL optimization

We introduce an RL optimization strategy that transforms the general-purpose Qwen3-32B into EduQwen 32B-RL1, a domain expert specializing in pedagogical knowledge. Our ablation studies compared Group Relative Policy Optimization (GRPO) (Shao et al., 2024) and Decoupled Advantage Policy Optimization (DAPO) (Liu et al., 2024). We found that DAPO's ability to decouple the policy update from the advantage estimation provided more stable gradients when training on the complex, multi-step reasoning chains required for pedagogical scaffolding, ultimately yielding superior performance on the CDPK Benchmark, and was therefore selected as our primary RL algorithm. In early ablation experiments evaluated at training step 20, the post-training evaluation accuracy was 85.43% for DAPO vs. 83.04% for GRPO under a matched training budget. Specifically, our implementation enhances training stability by employing the DAPO estimator for advantage computation and utilizing asymmetric clipping to stabilize policy updates. This asymmetric approach allows for a more robust exploration of high-reward reasoning paths while strictly maintaining a lower bound to prevent catastrophic policy divergence. Rather than training a dedicated reward model from scratch, we employed a Model-as-a-Judge framework in which a large open-source model (Qwen3-235B) evaluated the policy model's responses to pedagogy-dataset questions and produced the reward signal. The judge assessed responses against a functional-correctness criterion: a response was rewarded to the extent that it steered the policy model toward the correct resolution of the pedagogical item, thereby aligning training with guided-learning behavior rather than answer-giving alone. The pedagogical quality was operationalized through this automated functional criterion; at this stage, we did not involve human educators in generating reward labels. To assess the reliability of the automated rewards, we conducted manual spot-checks on a subset of the judge's decisions, which qualitatively confirmed that the judge model reliably distinguished valid, well-reasoned responses from flawed ones. Because the optimization objective targeted functional pedagogical correctness on the dataset rather than the stylistic conventions of any particular leaderboard, we did not observe the reward model inducing bias toward benchmark-specific answer formatting. Our RL training incorporated several novel strategic innovations to maximize performance on the CDPK Benchmark.

We implemented progressive training by difficulty level, beginning with simpler pedagogical questions and gradually increasing complexity as the model's performance improved. This curriculum learning approach (Bengio et al., 2009) allowed the model to build foundational pedagogical reasoning before tackling more nuanced teaching scenarios. Throughout training, we continuously increased the number of epochs, monitoring validation performance to determine optimal stopping points that balanced improvement against overfitting.

Our data processing strategy explicitly targeted the model's weaknesses through a rigorous failure-mode identification process. We first tested the Qwen3-32B base model on all questions in the pedagogy dataset, performing 30 attempts per question to identify items where the model exhibited uncertainty or incorrect reasoning. Questions answered correctly in all 30 attempts were excluded from training. The remaining questions were sorted by error frequency from lowest to highest, creating a difficulty-ordered curriculum of 440 training data points that progressed from easier to more challenging pedagogical scenarios. This carefully curated dataset formed the foundation for our first-stage RL optimization, ensuring that training focused precisely on the model's areas of greatest need. By training on these high-difficulty samples, the SFT phase acts as a corrective regularizer that smooths the policy's performance across the entire pedagogical domain and supports more consistent behavior across a broad range of teaching scenarios.

A critical innovation in our approach was training on “potentially incorrect questions”—examples where the model's initial responses were uncertain or showed reasoning flaws. By generating synthetic training data focused on these failure modes, similar to techniques in the Self-Taught Reasoner (STaR) (Zelikman et al., 2022), we enabled the model to strengthen its weakest areas iteratively. We also progressively increased rollout length from 5 to 8 steps during RL training, allowing the model to engage in longer chains of reasoning and better capture the multi-step nature of pedagogical decision-making. This innovative alignment process resulted in our RL-optimized model, EduQwen 32B-RL1, achieving 94.13% accuracy on the CDPK Benchmark, already establishing new SOTA performance for both open-source and proprietary models in educational pedagogy, and demonstrating strong performance on benchmarked pedagogical reasoning as a technical prerequisite for future investigation in applied educational settings.

Following the initial RL stage and subsequent SFT phase (described in Section 2.3.2), we applied an optional second round of RL optimization to further refine pedagogical reasoning capabilities. This second round of RL training utilized the same difficulty-ordered data points from the first RL stage, maintaining the progressive difficulty arrangement to reinforce the model's performance on challenging pedagogical items while building upon the improvements achieved through SFT.

#### 2.3.2 SFT

The difficulty-weighted data curation applied during RL effectively serves as an importance-sampling mechanism, where the synthetic data distribution is shifted toward the hard-negative space of the RL-optimized policy. Building on this targeted data distribution and following first-stage RL optimization, we apply a novel SFT strategy that converts EduQwen 32B-RL1 into an intermediate model (EduQwen 32B-SFT) during a second stage, further enhancing pedagogical performance and achieving new SOTA accuracy on the CDPK Benchmark. The intermediate model can optionally undergo a third-stage optimization with another round of RL (described in Subsection 2.3.3.) to produce EduQwen 32B-SFT-RL2, establishing SOTA accuracy performance on the CDPK benchmark for educational LLM applications. The SFT phase leverages the RL-trained model itself (EduQwen 32B-RL1) to synthesize high-quality training data, creating a self-reinforcing improvement loop. We prompted the RL-optimized model to generate 40,000 data points consisting of pedagogically sound responses to questions across the difficulty spectrum, then applied a rigorous filtering process to extract high-quality examples focused on the model's challenging areas.

A key innovation in our SFT approach was the strategic weighting of training examples by difficulty level, explicitly targeting high-difficulty pedagogical items that typical educational LLMs struggle with. Our data curation process applied gradient-based selection: we eliminated questions the model answered correctly consistently, retaining only items where the model exhibited uncertainty or errors. For questions with high baseline accuracy, we selected a single representative training example; for genuinely difficult questions with low accuracy, we retained all available high-quality examples to ensure comprehensive coverage of challenging pedagogical scenarios. This process yielded 1,050 high-quality, difficulty-ordered training data points that concentrated learning capacity on the model's areas of greatest need; this represents a retention rate of approximately 2.6%, reflecting the stringent quality and difficulty thresholds applied. We assigned increased training weights to high-difficulty questions, ensuring the model developed robust performance on the most challenging scenarios. This difficulty-aware training strategy helped address the typical performance degradation that models experience on complex edge cases and is particularly important for maintaining reliability on the kinds of challenging items that often contribute to uneven performance across assessment difficulty levels.

The SFT process utilized standard cross-entropy loss with the difficulty-weighted examples, training for multiple epochs while monitoring validation accuracy. We employed gradient accumulation and mixed-precision training to optimize computational efficiency. The combination of RL-generated synthetic data and difficulty-weighted training enabled the model to achieve a significant performance gain over the RL-only baseline. The outcome of this stage is EduQwen 32B-SFT, a pedagogical model that reaches new SOTA-level performance on the CDPK subset of The Pedagogy Benchmark, and whose performance can be further improved by applying an optional second round of RL to obtain our highest-reported SOTA accuracy within this benchmarked pedagogical setting.

#### 2.3.3 Second round RL optimization

Following the first round of RL optimization and subsequent SFT phase, we optionally apply a second round of RL optimization starting from EduQwen 32B-SFT and using the same difficulty-aware dataset constructed during the first RL round. Crucially, this final RL phase reuses the initial difficulty-aware dataset, allowing the model to leverage its refined knowledge to finally solve the problems it initially found challenging. This cyclical process of reinforcement, refinement, and re-reinforcement ensures a systematic improvement in the model's performance on complex pedagogical tasks, yielding our final model EduQwen 32B-SFT-RL2 as a high-performing pedagogical LLM suitable for further investigation in applied educational contexts.

The complete multi-stage optimization procedure—comprising the initial RL stage for hard-negative identification, the difficulty-weighted SFT phase for knowledge distillation, and the optional final RL refinement—is formalized in Algorithm 1. This framework illustrates the iterative transition toward domain expertise, specifically detailing how we stabilize policy updates across all phases by employing the DAPO estimator and expansive asymmetric clipping, thereby providing a replicable technical blueprint for constructing pedagogically specialized, open-source LLMs.

Algorithm 1 **Pedagogy domain training via RL-SFT-RL.**

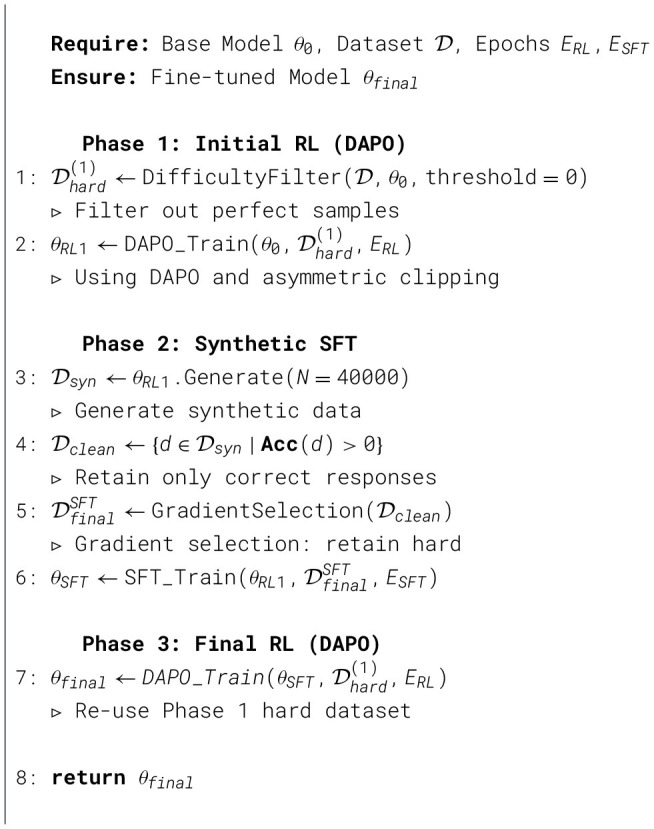

## 3 Results

Our innovative multi-stage optimization strategy—RL to produce EduQwen 32B-RL1 followed by strategic SFT to obtain EduQwen 32B-SFT, and optionally a second RL round that yields EduQwen 32B-SFT-RL2—results in strong performance on the CDPK Benchmark, establishing new SOTA results on the CDPK subset of The Pedagogy Benchmark leaderboard as of March 2026. EduQwen 32B-RL1 reaches 94.13% accuracy, EduQwen 32B-SFT reaches 96.20% accuracy, and with the optional second RL round, EduQwen 32B-SFT-RL2 reaches 96.52% accuracy, with all three models achieving higher accuracy on CDPK than any open-source or proprietary model reported on The Pedagogy Benchmark Leaderboard snapshot as of March 2026, including the previous best-performing Gemini-3 Pro at 90.55%. Table 1 showcases the performance of baseline models and our optimized variants using a common, publicly documented benchmark for comparability and transparency.

**Table 1:** Performance of EduQwen variants (EduQwen 32B-RL1, EduQwen 32B-SFT, EduQwen 32B-SFT-RL2) on the CDPK Benchmark compared with baseline and leaderboard models.

Model	Inference-time compute	Replicated accuracy (95% CI) (%)	Reported accuracy (%)
Gemini-3Pro (Rk #1)	Benchmark leader	–	**90.55**
Qwen3-235B-A22B-Thinking (Rk #19)	Random few-shot	86.63 [84.41, 88.85]	85.65
Qwen2.5-72B-Instruct (Rk #66)	Random few-shot	78.60 [75.92, 81.28]	75.42
Qwen3-32B Base Model (Rk #28)	Random few-shot	83.37 [80.83, 85.64]	82.42
**EduQwen 32B-RL1**	RL 1st round	**94.13 [92.42, 95.47]**	–
**EduQwen 32B-SFT**	SFT	**96.20 [94.76, 97.25]**	–
**EduQwen 32B-SFT-RL2**	RL 2nd round	**96.52 [95.13, 97.53]**	–

**Bold** font indicates the best performance between our EduQwen models and the Cross-Domain Pedagogical Knowledge (CDPK) leader (as of March 2026). **Red** font indicates the previous SOTA benchmark leader prior to this work. CI, confidence interval; Rk, rank; RL, reinforcement learning; SFT, supervised fine-tuning. Reported accuracy refers to the performance published on the CDPK Benchmark Leaderboard, while replicated accuracy indicates our experimental results.

The performance of EduQwen 32B-RL1, EduQwen 32B-SFT, and EduQwen 32B-SFT-RL2 in comparison to other top-performing models on the CDPK Benchmark Leaderboard (AI for Education, 2026) demonstrates that our approach establishes a new SOTA on the CDPK pedagogical knowledge benchmark, as reported on the interactive leaderboard snapshot we consider. The substantial improvement from the Qwen3-32B baseline (83.37%) to our final model (96.52%) represents a 13.15 point gain, showcasing the effectiveness of our innovative combined RL and SFT optimization pipeline for enhancing benchmarked pedagogical reasoning performance in an open-source, mid-sized model. The performance gains demonstrated by all three optimized model tiers—EduQwen 32B-RL1 (94.13%), EduQwen 32B-SFT (96.20%), and EduQwen 32B-SFT-RL2 (96.52%)—over the Qwen3-32B baseline (83.37%) are highly statistically significant, with each comparison yielding a two-tailed *p* < 0.001 (McNemar's test).

**Figure 1:**
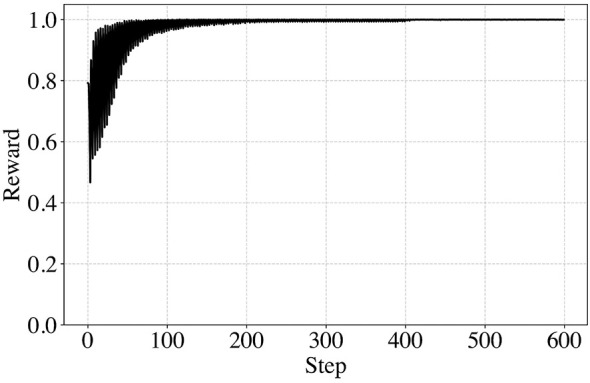
Reward curve during first-stage reinforcement learning optimization of EduQwen 32B-RL1, illustrating convergence across training iterations.

Figure 1 illustrates the training dynamics of the RL optimization phase. The reward signal exhibits high variance during early training as the model explores the pedagogical reasoning space, then shows rapid improvement with reward climbing from approximately 0.5 to near 1.0 within the first 100 steps. Convergence is achieved by approximately step 400, with variance decreasing substantially as the model stabilizes on pedagogically-aligned response patterns that are rewarded by the CDPK-centric optimization process.

**Figure 2:**
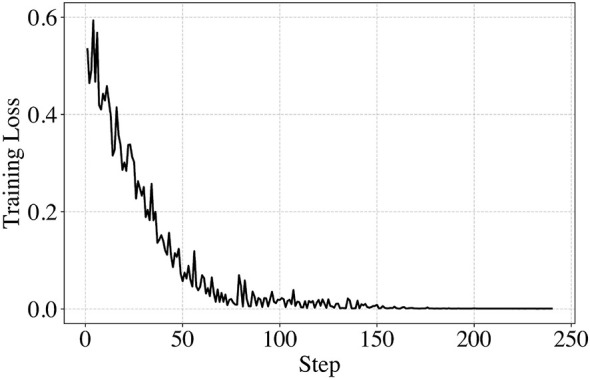
Training loss curve during supervised fine-tuning of EduQwen 32B-SFT using synthetic data generated by the RL-optimized model.

Figure 2 shows the corresponding SFT training loss curve, which decreases from approximately 0.5 to near zero, with convergence occurring by approximately step 150. The rapid convergence of both training phases suggests that the Qwen3-32B base model is highly amenable to pedagogical specialization, and that our difficulty-weighted curriculum provides an efficient learning signal for concentrating model capacity on challenging pedagogical items.

Notably, all stages of our optimization contributed meaningfully to the final performance: the first RL stage provided a 10.76 percentage point improvement over baseline (83.37% to 94.13%), the subsequent SFT phase added an additional 2.07 points (94.13% to 96.20%), and the optional second RL round contributed a further 0.32 points (96.20% to 96.52%). Experimental records confirm that both RL and SFT processes reached complete convergence (Figures 1, 2) within the scope of our training and validation setup.

This demonstrates that our multi-stage approach captures complementary aspects of pedagogical reasoning—RL for strategic alignment and SFT for knowledge refinement. Critically, our optimization strategy applied to the dense 32-billion parameter model surpasses the performance scores of significantly larger models such as Qwen3-235B-A22B-Thinking (86.63%) and Qwen2.5-72B-Instruct (78.60%) reported as of March 2026, demonstrating that domain-specialized optimization substantially improves benchmarked pedagogical performance in smaller open-source models that are more cost-efficient than running very large models with broad general knowledge and therefore particularly attractive for scenarios where computational budget is a key constraint in educational deployment. A practical illustration of this cost profile is provided in the Supplementary material. Once trained, EduQwen 32B-SFT-RL2 can be served on a single high-memory GPU, and at representative public cloud rates the estimated per-query inference cost is on the order of a fraction of a cent. By comparison, answering the same queries through a proprietary API such as Gemini-3 Pro would incur recurring per-token charges that, at scale and over time, substantially exceed the one-time cost of optimizing and self-hosting an open model. While such estimates depend on factors such as usage volume, hardware, and current pricing, they support the broader argument that a self-hosted, domain-specialized open model can offer a favorable cost structure for sustained, high-volume educational use.

## 4 Discussion

Our results demonstrate that the EduQwen 32B-RL1, EduQwen 32B-SFT, and optional EduQwen 32B-SFT-RL2 pipeline achieves substantial improvements in LLM performance on specialized pedagogical tasks in education, establishing new SOTA performance on the CDPK Benchmark as reported on the interactive Pedagogy Benchmark leaderboard as of March 2026. The 96.52% accuracy achieved by our final model (EduQwen 32B-SFT-RL2) is the highest CDPK accuracy reported on that leaderboard snapshot, exceeding the contemporaneous Gemini-3 Pro entry by 5.97 percentage points under the respective evaluation conditions. These results support the development of widely accessible, transparent, and high-performing models for benchmarked pedagogical knowledge that can be realistically considered by institutions with constrained computational and financial resources. It should be noted, however, that strong benchmark performance on pedagogical knowledge questions does not itself constitute evidence of effective classroom tutoring; actual educational impact depends on teacher judgment, learner epistemic beliefs, the fostering of critical inquiry, and sustained deployment in authentic instructional contexts (Rind, 2026; Rind et al., 2026).

The effectiveness of our novel multi-stage optimization pipeline highlights several important insights for domain-specific LLM development in educational applications. First, RL training with pedagogically-aligned reward models appears to shift model behavior toward guided-learning-like response patterns on CDPK-style pedagogical items rather than direct answer provision. This aligns with the theoretical expectation that reward shaping toward process-level feedback, as opposed to answer confirmation, produces models whose response patterns more closely mirror the scaffolding behavior described by Hattie and Vygotsky (Hattie and Timperley, 2007; Vygotsky, 1978), suggesting that the reward model successfully encoded a meaningful pedagogical signal rather than merely optimizing for surface-level correctness. Second, training on “potentially incorrect questions” and using progressive difficulty curricula enables targeted improvement on model weaknesses. This outcome is consistent with curriculum learning theory (Bengio et al., 2009), which predicts that ordering training examples by difficulty accelerates convergence and improves generalization by allowing the model to build robust representations before encountering edge cases. Third, leveraging the RL-optimized model to generate synthetic training data for SFT creates a virtuous cycle that pushes performance beyond what either technique achieves independently in this benchmarked pedagogical setting. This self-reinforcing dynamic is particularly significant because it suggests that the RL-trained model has internalized a sufficiently robust pedagogical reasoning strategy to generate reliable training signal for its own subsequent refinement; this is a property that distinguishes genuine domain adaptation from surface-level benchmark optimization. Fourth, the strategic weighting of synthetic training data by difficulty during the SFT phase acts as a critical safeguard against catastrophic forgetting on complex edge cases. While standard SFT often dilutes a model's performance on highly nuanced tasks by averaging learning across all examples, assigning higher training weights to difficult items forces the model to allocate more representational capacity to the most challenging pedagogical scenarios, such as navigating stubborn learner misconceptions. This ensures that the model maintains its robust scaffolding capabilities even after the explicit reward constraints of the initial RL phase are removed.

The combination of strategic innovations during RL training—including rollout step adjustment (e.g., increased rollout length from 5 to 8 steps), the selection of DAPO over GRPO, progressive difficulty curricula, and continuously increasing training epochs—proved collectively valuable for pedagogical reasoning performance, which often requires multi-step consideration of learner needs, appropriate scaffolding, and strategic information revelation. This interpretation is consistent with the multi-step nature of PCK as described by Shulman (1987): effective pedagogical responses require the model to simultaneously consider the learner's current knowledge state, identify the likely source of confusion, select an appropriate representational strategy, and sequence information revelation accordingly, which is a reasoning chain that cannot be compressed into short rollouts without loss of instructional quality. This finding suggests that pedagogical tasks benefit from holistic optimization strategies that enable longer reasoning chains and targeted learning without requiring very large, compute-intensive frontier models. This finding also speaks to a broader principle in domain-specific AI development: pedagogical knowledge, as operationalized by the CDPK Benchmark, is a specialized reasoning capacity that benefits more from targeted alignment than from scale alone. General-purpose frontier models, despite their breadth, are not optimized for the specific inferential patterns that characterize expert pedagogical reasoning (Shulman, 1986), which may explain why domain-specialized mid-sized models can exceed their performance on benchmarked pedagogical tasks.

Our optimization strategy applied to a dense 32-billion parameter open-source model (Qwen3-32B) surpasses the performance of significantly larger models reported as of March 2026, including those exceeding 200 billion parameters. This finding has direct implications for educational applications of machine learning: domain-specialized, mid-sized open-source models such as EduQwen 32B-RL1, EduQwen 32B-SFT, and EduQwen 32B-SFT-RL2 can deliver expert-level performance on benchmarked pedagogical knowledge tasks at a fraction of the cost of very large proprietary systems. The open-source architecture of our model directly addresses the need for algorithmic accountability and transparency, enabling educators to audit for bias and customize the system for diverse cultural and linguistic learning contexts; these capabilities are unavailable with proprietary systems. These characteristics make our approach particularly promising for real-world educational deployment (pending validation in authentic instructional contexts), across diverse socioeconomic contexts with equity-oriented goals, where institutional constraints and infrastructure limitations often preclude reliance on expensive proprietary APIs and where technically strong, open models are a prerequisite for narrowing the access gaps. Framed through the lens of cognitive load theory, EduQwen is best understood as a tool that redistributes rather than replaces teacher cognitive work, handling routine knowledge retrieval and scaffolded explanation so that educators can direct their expertise toward higher-order instructional decisions, relationship-building, and adaptive responses to individual learner needs (Rind, 2026).

To assess the generalizability of our optimization approach beyond the CDPK Benchmark, we conducted preliminary experiments on TutorBench (Srinivasa et al., 2025), a recently introduced multimodal benchmark that evaluates LLM tutoring capabilities across both text-only and multimodal educational scenarios. Using Qwen3-30B-VL as a vision-language base model—selected for its vision-language capabilities required by TutorBench's multimodal tasks—we applied a single SFT optimization run using 596 high-quality synthetic responses filtered from model outputs. As shown in Table 2, our SFT-optimized model (EduQwen 30B-VL-SFT) achieves 61.64% overall accuracy, which is higher than the 58.52% reported for Gemini-3 Pro (Rank no. 3) on the TutorBench leaderboard snapshot we consider (scale.com, 2025), despite starting from a weaker baseline (55.39%). These preliminary results suggest that our difficulty-weighted SFT methodology transfers effectively across educational benchmarks and modalities and can be extended beyond a single benchmark or modality, although direct rank comparisons should be interpreted cautiously because our evaluation used Claude-4.5-Sonnet as judge model, whereas the official leaderboard uses Claude-4-Sonnet, and judge differences may affect absolute scores; the effect of this judge discrepancy on scores is unknown and represents a limitation of the cross-leaderboard comparison.

**Table 2:** Performance of EduQwen 30B-VL-SFT on the TutorBench Benchmark, compared with baseline and leaderboard models, as reported on the TutorBench leaderboard snapshot we reference.

Model	Judge model	Text-only (%)	Multimodal (%)	Overall (%)	TutorBench (%)
Gemini-2.5-pro-preview-06-05 (Rk#1)	Cl-4-Sonnet	–	–	–	**55.65**
Gemini-3-pro-preview (Rk#3)	Cl-4-Sonnet	–	–	–	53.67
Gemini-3Pro	Cl-4.5-Sonnet	58.41	58.67	58.52	–
Qwen3-30B-VL (Base Model)	Cl-4.5-Sonnet	60.21	51.53	55.39	–
**EduQwen 30B-VL-SFT**	Cl-4.5-Sonnet	**64.10**	**59.40**	**61.64**	–

**Bold** font indicates the best performance between our EduQwen model and the TutorBench reference models listed in this table. **Red** font indicates the previous benchmark leader (as of March 2026) prior to this work. Rk, rank; Cl, Claude; SFT, supervised fine-tuning.

While our current approach achieves new SOTA performance, several opportunities exist for future work. Exploring alternative RL algorithms and optimization approaches may offer efficiency gains or performance improvements that further reduce the resource requirements for strong pedagogical models.

A key limitation of our study is that all primary results are obtained on teacher-exam-style multiple-choice questions from CDPK, so future work should examine free-form tutoring dialogs and longitudinal learning gains to fully validate pedagogical impact. The reported gains are likewise best understood as evidence of in-distribution pedagogical performance, and we identify out-of-distribution and fully held-out evaluation as a further direction for future work. In particular, evaluating EduQwen models in interactive classroom or platform settings would help determine whether benchmark gains translate into sustained improvements in real learners' reasoning and outcomes, and whether the technical advantages we demonstrate can support more consistent access to high-quality guidance across diverse learner populations.

Our work demonstrates that open-source models can not only match but also exceed proprietary models on specialized tasks when equipped with appropriate optimization strategies. This finding has critical implications for efforts to deploy educational AI in more equitable ways globally, where transparency, customizability, cost-effectiveness, and institutional sovereignty are paramount for closing digital divides. The ability to fine-tune open-source models for specific pedagogical frameworks while maintaining full control over model behavior and data addresses key equity concerns about deploying AI in diverse educational settings, particularly in under-resourced contexts where reliance on proprietary systems reinforces technological dependencies and constrains local control over educational technologies.

## 5 Conclusion

In this study, we demonstrate that a novel multi-stage optimization approach combining RL and SFT yields EduQwen 32B-RL1, EduQwen 32B-SFT, and EduQwen 32B-SFT-RL2, which are open-source models optimized for benchmarked pedagogical knowledge that achieve strong performance on standardized educational assessments, establishing the new SOTA on the CDPK Benchmark and related pedagogical evaluations. Our final model achieves 96.52% accuracy on the CDPK Benchmark, the highest performance among all open-source and proprietary models currently listed on The Pedagogy Benchmark Leaderboard. The combination of RL-based alignment with pedagogical reasoning, progressive difficulty training, focus on challenging examples, and difficulty-weighted SFT created a powerful optimization pipeline that substantially advances benchmarked pedagogical knowledge in a dense 32-billion parameter model into a SOTA-level pedagogical knowledge system on the CDPK Benchmark. Our results demonstrate that domain-specialized smaller open-source models can surpass the reported performance (March 2026) of significantly larger general-purpose systems on pedagogical knowledge benchmarks, while offering superior cost-efficiency while maintaining the critical advantages of transparency, customizability, and adaptability, which are important considerations for equitable and responsible development of educational AI, with real-world deployment effectiveness dependent on teacher integration, learner context, and sustained use in authentic instructional settings. This concept has future application potential across diverse global educational contexts, including under-resourced institutions that require technically strong yet practically deployable agentic educational AI. Several concrete directions for future research follow from this work. First, future work should explore whether the same optimization pipeline can achieve comparable gains when applied to smaller open-source architectures, such as 7B or 14B parameter models, which would further reduce the computational requirements for potential deployment in the most resource-constrained educational settings. Second, the difficulty-weighted SFT methodology demonstrated here warrants evaluation on additional educational benchmarks and subject domains beyond the CDPK Benchmark, to assess the generalizability of the approach. Third, future work should investigate whether free-form tutoring dialogue, rather than multiple-choice benchmarks, can serve as both a training and evaluation signal, bringing the optimization target closer to authentic pedagogical interaction. Fourth, longitudinal studies examining learning gains in students interacting with EduQwen over sustained periods would provide the real-world validation that benchmark performance alone cannot supply. Ultimately, translating these technical gains into genuine pedagogical impact will require situating EduQwen within real classroom contexts, examining how these tools successfully redistribute teachers' cognitive load, and supporting the development of learners' critical epistemic engagement with AI-generated guidance (Rind, 2026; Rind et al., 2026).

## Data Availability

The raw data supporting the conclusions of this article will be made available by the authors, without undue reservation.
