# Supplementary material for: Application-driven pedagogical knowledge optimization of open-source LLMs via reinforcement learning and supervised fine-tuning
Source: Front Artif Intell. 2026 Jul 1;9:1851993. doi: 10.3389/frai.2026.1851993 (PMC13369524; doi:10.3389/frai.2026.1851993)
Supplement: Supplementary file 1 [file Supplementary_file_1.pdf]

1 SUPPLEMENTARY MATERIAL: TECHNICAL IMPLEMENTATION DETAILS

1.1 Training Hyperparameters

The two reinforcement learning stages (first-stage RL producing EduQwen 32B-RL1 and second-stage RL producing EduQwen 32B-SFT-RL2) used identical hyperparameter configurations, summarized in Table 3. The supervised fine-tuning stage configuration is summarized in Table 4.

Table 3. Reinforcement learning (RL1 and RL2) training hyperparameters.

Parameter	Value
Algorithm	DAPO (decoupled advantage policy optimization)
Advantage estimator	GRPO-style group-relative
Total training epochs	240
Learning rate	1×10^{-6}
Total training batch size	64
PPO mini-batch size (per node)	32
Micro-batch size per GPU	1
Rollout responses per prompt (n)	16
Gradient clipping	0.5
Clip ratio (default)	0.2
Clip ratio low	0.4
Clip ratio high	0.5
Loss aggregation mode	token-mean
Loss mode	geometric mean
KL in reward	Disabled
KL loss coefficient	0.0 (actual; script default 0.001)
KL loss type	low-variance KL
FSDP parameter offload	Enabled
FSDP optimizer offload	Enabled (fraction 1.0)
Gradient checkpointing	Enabled

Table 4. Supervised fine-tuning (SFT) training hyperparameters.

Parameter	Value
Epochs	30 (240 global steps)
Per-device batch size	1
Gradient accumulation steps	16
Effective batch size	128
Learning rate	1×10^{-5}
LR scheduler	Cosine
Warmup ratio	0.05
Optimizer	AdamW (fused)
Adam β	(0.9, 0.95)
Weight decay	0.1
Max gradient norm	1.0
Precision	BF16
Loss	Cross-entropy (difficulty-weighted)
Gradient checkpointing	Enabled
DeepSpeed	ZeRO Stage 3, CPU offload (optimizer + parameters)

1.2 Hardware and Compute Resources

All training was conducted on NVIDIA H200 GPUs (141 GiB VRAM per GPU). The reinforcement learning stages used two nodes of 8 GPUs each (16 GPUs total), connected via a multi-node Ray and vLLM configuration with an NCCL backend. The supervised fine-tuning stage used a single 8-GPU node. Total compute across all stages was approximately 10,560 GPU-hours, distributed as summarized in Table 5.

Table 5. Compute resources by training stage.

Stage	Hardware	GPU-hours
RL1 (first-stage RL)	16× H200 (2 nodes)	5,120
SFT	8× H200 (1 node)	320
RL2 (second-stage RL)	16× H200 (2 nodes)	5,120
Total	–	∼10,560

1.3 Evaluation and Synthetic Data Generation Prompts

This subsection provides the verbatim prompts used during model evaluation and synthetic data generation. The evaluation prompt was used to judge whether a model's response to a multiple-choice question matched the answer key. Placeholders shown in braces ({question}, {correct_answer_letter}, {model_response}) were populated at runtime.

Evaluation prompt.

```
You are an objective evaluator. I will provide a multiple-choice question,
    the correct answer key, and a student's response. Your task is to
   determine if the student's response matches the correct answer.

--- Question ---
{question}

--- Correct Answer Key ---
{correct_answer_letter}

--- Student's Response ---
{model_response}

--- Instruction ---
1. Ignore the student's reasoning process if provided.
2. Focus on their final conclusion/choice.
3. If the student selects option {correct_answer_letter}, output 'CORRECT'.

4. If the student selects a different option or fails to choose, output '
   WRONG'.
5. Output ONLY 'CORRECT' or 'WRONG' without any explanation.
```

Synthetic data generation prompt.

```
You are an expert AI trainer specializing in reasoning models. I need you
   to generate a new training example.

--- REFERENCE ORIGINAL QUESTION ---
Category: General
Question: {question}

TASK:
1. Create a NEW, SIMILAR multiple-choice question (same difficulty,
   different scenario).
2. Provide the 4 options and the correct answer.
3. CRUCIAL: Write the 'detailed_analysis' in a specific Reason-then-Answer
    format.
  - The analysis MUST start with the tag <think>.
  - Inside <think>...</think>, write the step-by-step internal reasoning
     process a student should go through.
  - After </think>, state the final conclusion clearly.

Output MUST be valid JSON strictly following this structure:
{
  "new_question": "...",
  "option_a": "...",
  "option_b": "...",
  "option_c": "...",
  "option_d": "...",
  "correct_answer": "A/B/C/D",
  "detailed_analysis": "<think>First, I need to analyze the student's
     behavior... [reasoning process] ...</think> Therefore, the correct
     answer is B."
}
```

1.4 Filtering Criteria and Validation Procedure

Synthetic data filtering. The first-stage RL model (EduQwen 32B-RL1) was prompted to generate 40,000 candidate synthetic training examples spanning the difficulty spectrum. These candidates were filtered using a model-based judge (see Section 2.3.1), which scored each example for correctness and quality; only high-scoring examples whose responses were verified as being correct were retained. Examples the base model answered correctly with high consistency were down-weighted to a single representative instance, while genuinely difficult items with low baseline accuracy were retained in full to ensure comprehensive coverage of challenging pedagogical scenarios. This gradient-based selection process yielded 1,050 high-quality, difficulty-ordered training examples, corresponding to a retention rate of approximately 2.6%.

Validation and stopping criteria. During RL training, the reward signal produced by the reward model was monitored to confirm that training was proceeding normally and converging in a stable manner.

Training was terminated after completing 240 epochs. Following training, each model checkpoint was evaluated on the CDPK pedagogy test split, with a model-based judge determining the correctness of each response against the ground-truth answer key.

1.5 Inference Parameters

Inference was served using the vLLM engine in two distinct contexts: rollout generation during RL training, and final model evaluation. The two contexts used different sampling settings, reflecting their different purposes; exploration during training versus deterministic scoring during evaluation. Both configurations are summarized in Table 6.

Table 6. Inference and sampling parameters for RL rollout generation and final evaluation.

Parameter	RL Rollout	Evaluation
Inference engine	vLLM	vLLM
Tensor parallelism	8 (per node)	8 (per node)
Max prompt length	2,048 tokens	2,048 tokens
Max response length	8,192 tokens	512 tokens
Temperature	1.0	0.0
Top-p	1.0	0.7
Top-k	-1 (disabled)	-1 (disabled)
Chunked prefill	Disabled	Disabled
Enforce eager	Disabled	Disabled
Free cache engine	Disabled	Disabled
Filter overlong prompts	Enabled	Enabled
Truncation mode	Error on overflow	Error on overflow

1.6 Cost and Deployment Analysis

This subsection provides an approximate cost comparison to contextualize the cost-efficiency claims in the main text. All figures are illustrative estimates based on representative public pricing at the time of writing and will vary with hardware, usage volume, and prevailing rates.

Training cost. The full multi-stage pipeline consumed approximately 10,560 GPU-hours on NVIDIA H200 hardware (Appendix 1.2). At representative on-demand cloud rates, this corresponds to a one-time optimization cost of roughly $35K–$40K, after which the resulting model can be reused indefinitely without further training expenditure.

Inference and deployment. The trained 32B model can be served for inference on a single high-memory GPU (for example, one H200, or comparable hardware with sufficient VRAM, optionally with quantization to reduce the memory footprint). With a typical query consuming on the order of a few thousand input tokens and a few hundred output tokens, the estimated self-hosted inference cost is approximately $0.001–$0.002 per query.

Comparison with proprietary APIs. Serving equivalent queries through a proprietary API incurs recurring per-token charges. Using published Gemini-3 Pro token pricing as a reference point, the corresponding per-query cost is on the order of $0.01—several times the estimated self-hosted cost—and these charges recur for every query in perpetuity. For institutions anticipating sustained, high-volume usage, the one-time cost of optimizing and self-hosting an open model can therefore be recovered over time relative to ongoing API expenditure, while also preserving data control and deployment autonomy.
